# Impact of the Physicochemical Composition and Microbial Diversity in Apple Juice Fermentation Process: A Review

**DOI:** 10.3390/molecules25163698

**Published:** 2020-08-13

**Authors:** Marina Al Daccache, Mohamed Koubaa, Richard G. Maroun, Dominique Salameh, Nicolas Louka, Eugène Vorobiev

**Affiliations:** 1 Sorbonne University, Université de technologie de Compiègne, ESCOM, EA 4297 TIMR, Centre de recherche Royallieu, CEDEX CS 60319, 60203 Compiègne, France; marina.daccache@net.usj.edu.lb (M.A.D.); eugene.vorobiev@utc.fr (E.V.); 2 Laboratoire CTA, UR TVA, Centre d’Analyses et de Recherche, Faculté des Sciences, Université Saint-Joseph, Beyrouth 1104 2020, Lebanon; richard.maroun@usj.edu.lb (R.G.M.); dominique.salameh@usj.edu.lb (D.S.); nicolas.louka@usj.edu.lb (N.L.); 3 ESCOM, UTC, EA 4297 TIMR, 1 allée du réseau Jean-Marie Buckmaster, 60200 Compiègne, France

**Keywords:** apples, cider, fermentation, physicochemical composition, microorganisms

## Abstract

Fermented apple beverages are produced all over the world with diverse characteristics associated with each country. Despite the diversifications, cider producers are confronted with similar issues and risks. The nature of the raw material, also known as the fermentation medium, plays a key role in fermentation. A well-defined composition of apples is, therefore, required to produce cider with good quality. In addition, ferment and its metabolism are important factors in the fermentation process. The producers of cider and other alcoholic beverages are looking in general for novel yeast strains or for the use of native strains to produce “authentic” and diversified beverages that are distinct from each other, and that attract more and more consumers. Research articles on cider production are infrequent compared to wine production, especially on the impact of the chemical composition and microbial diversity of apples on fermentation. Even though the processing of fermented beverages is close in terms of microbial interactions and production, the study of the specific properties of apples and the production challenges of cider production is advantageous and meaningful for cider producers. This review summarizes the current knowledge on apple composition and the impact of the must composition on fermentation and yeast growth. In addition, the microbial diversity of cider, activities, and its influence on fermentation are reviewed.

## 1. Introduction

Apples represent a very particular fruit known for their unique symbolic richness over time. Later, the different studies proved the importance of that fruit due to its chemical composition and specifically its antioxidant characteristics. The fruit belongs to the “*Maloideae*” subfamily and to the “*Rosacea*” family. It represents one of the most important deciduous tree fruits that are generally grown in temperate and tropical regions [1]. Apple is one of the most produced and consumed fruits in the world. It is ranked as the third-most fruit produced worldwide after bananas and watermelon with a production that reached 75 million tons in 2018–2019 [2]. China stands as the largest producer with increasing production of almost 33 million tons per year, followed by the European Union (EU) producing 15 million tons per year (Table 1). The United States comes in the third rank, producing 5.6 million tons of apples in 2019. The main apple producers in Europe are Poland, France, and Italy. Turkey and Iran produce around 3 million tons per year each while the production of Chile, Russia, Ukraine, and Brazil is around 1.2 million tons per year (Table 1).

Furthermore, apple juice is the main raw material for several beverages’ production. Vinegar, cider, calvados, and apple wine are obtained from apple juice fermentation, depending on the conditions applied. This review focuses on the alcoholic fermentation of apple juice to produce cider. Over the past years, different definitions were accorded to the word “fermentation”. The term was first applied to describe the production of wine and specifically the bubbling caused by the production of carbon dioxide. Nowadays, alcoholic fermentation is known as a biological complex process where yeasts convert sugars like glucose, fructose, and sucrose into cellular energy, ethanol, carbon dioxide, and other metabolic byproducts. Different parameters may affect the fermented product such as the composition of the raw materials, the microorganism used during the fermentation, and the process parameters and conditions.

## 2. Physicochemical Composition and Microbial Diversity of Apple Fruits

### 2.1. Fresh Apple Composition

Mature apples are mainly composed of about 85% water, 12–14% carbohydrates, 0.30–1% organic acid, 0.30% proteins, and less than 0.10% lipids, minerals, and vitamins [3]. The variation in the biochemical composition is mainly related to the variety and maturity, as well as the agronomic and climatic conditions. Furthermore, the soil composition may have a great impact on the fruit quality and composition. For example, nitrogen has a significant impact on fruit size and composition. High levels of nitrogen in apples may reduce the amount of soluble solids and raise the titratable acidity [4]. In addition, potassium intensifies the fruit color and significantly decreases the fruit firmness. Enough potassium concentration increases the apple sugar content, improves the fruit quality, the surface color, aroma, flavor, and storability [5]. Furthermore, calcium can be positively associated with fruit firmness and prevents the fruit from softening during storage [6,7]. Boron is also an essential trace element for the normal growth and development of apples.

#### 2.1.1. Sugars

The two main monosaccharides in apples are glucose and fructose, whereas sucrose is the main disaccharide (Table 2). D-sorbitol, in the form of alcoholic sugar, is abundant in apples with a concentration of 300 to 800 mg/100 mL of apple juice [8]. In addition, all fruits contain cellulose, hemicellulose (pentosans), and pectin.

#### 2.1.2. Organic Acids

L-malic acid is the main organic acid found in apples. Citric acid is found in very low concentrations, with thousands of times lower concentration than that of malic acid. The apple contains at least nine to 12 acids in small fractions. The acidity of apples can vary from 0.1 to 2.5 g of malic acid per 100 g of juice [10]. In addition to malic acid, citric, succinic, citramalic, shikimic, glyceric, glyoxylic, isocitric, glycolic, lactic, and galacturonic acids are present in apple juice. Three different keto acids have been found: Oxaloacetic acid, pyruvic acid, and ketoglutaric acid. However, these acids represent only a small fraction of the total organic acids present.

#### 2.1.3. Phenolic Compounds

The measurement of the phenolic substances present in the fruit has been of great interest to pomologists and cider producers (Table 3). Phenolic compounds, which are secondary plant metabolites, play a major role in the sensory, nutritional, and antioxidant properties of the fruit. Structurally, polyphenols are all composed of one or more aromatic rings with different structures that allow their classification into categories. Phenolic compounds are distinguished by the number and sequence of aromatic rings, the number and position of hydroxyl groups, and the presence of nonphenolic substitutes like, for example, alkyl groups, sugars, and organic acids. The phenolic compounds found in apples fall into two categories: Phenolic acids and flavonoids.

##### Phenolic Acids

Hydroxycinnamic acid esters represent one of the main classes of phenolic acids in different apple varieties [12]. Hydroxycinnamic acid is found in its esterified form. Chlorogenic acid, an ester of caffeic and quinic acid, is present in relatively high concentrations in both the peel and flesh of most apple cultivars. Chlorogenic acid oxidation is mainly responsible for the browning occurring in apple juice and cider [13]. P-coumaroylquinic acid is also found in apple fruit, but in lower concentrations [12].

##### Flavonoids

Flavonoids are widely found in different fruit and vegetable tissues such as leaves, seeds, bark, and flowers [14]. Flavonoids’ skeleton is composed of diphenylpropane (C6-C3-C6) with the three-carbon bridge between the phenyl groups connected with oxygen [15]. Thirteen subgroups of flavonoids exist according to differences in the number of substituted hydroxyl groups, degree of unsaturation, and degree of oxidation of the three-carbon bridge [15,16]. Flavonoids are referred to as glycosides when they contain one or more sugars and as aglycones when no sugar group is present [17]. In apple, anthocyanidin, flavanol (also named flavan-3-ols), flavonols (mainly quercetin glycosides), and dihydrochalcones are the major subgroups of flavonoids [18,19].

##### Flavan-3-ols

Flavan-3-ols’ class is the only class of flavonoids found in plants under their aglycone form. The skeleton of the base unit is represented by the flavane nucleus. It is a very large family, constituted of 11 subclasses. Flavan-3-ols exist in the monomeric or polymeric form. The monomeric flavan-3-ols constitute the second-largest class of polyphenols in apple fruit after hydroxycinnamic acids. They are represented only by (+)-catechin (CAT) and (-)-epicatechin (EC). The (-)-epicatechin is always predominantly present in apples with concentrations ranging from 46 mg.kg^−1^ to 2225 mg.kg^−1^ in Golden Delicious. The (+)-catechin is a minor flavanol, of which the concentrations may vary from 6 mg.kg^−1^ in Broxwood Foxwhelp to 408 mg.kg^−1^ in Yarlington Mill in the flesh [19,20].

##### Flavonols

Flavonols are commonly found in all fruits as 3-glycosides. Quercetin, an efficient antioxidant, is found in high concentrations of 49 mg/kg of apples [8]. Flavonols are usually yellow compounds that are responsible for the yellow color of certain apples’ epidermis.

##### Dihydrochalcone

Dihydrochalcones represent a specific class of flavonoids found in apple fruits. The aglycone dihydrochalcone is phloretin found only in a glycoside form in the fruit. Phloridzin (PLZ, phloretin glucoside) and phloretin xyloglucoside (XPLT) are the most widely described glycosides in the literature [19,21]. This family is particularly concentrated in pips. They can thereby represent more than 3 g/kg, i.e., 66% of the present phenolic compounds [19].

#### 2.1.4. Lipids

Apple fruits have commonly low lipid content, ranging from 0.1 to 0.5% of fresh weight. High levels of lipids are usually detected in the fruit seeds. The lipid fraction in fruits is composed of triacylglycerols, glycolipids and phospholipids, carotenoids, triterpenoids, and waxes. The lipid content in the apple fruits is detailed in Table 4.

#### 2.1.5. Vitamins

Vitamin C is biosynthesized in plants from hexoses such as glucose. Vitamin C content, also known as L-ascorbic acid, is ranging from 3 to 35 mg/100 g of the edible portion of the apple. It has a very high antioxidant activity. Vitamin B12, vitamin D, and tocopherols are found in trace amounts [8].

#### 2.1.6. Minerals

Minerals, also called inorganic nutrients, are found in all fruits. The most important cation and anion are potassium and phosphorus, respectively. Other elements such as sodium, calcium, and iron are also present at lower concentrations (Table 5).

### 2.2. Microbial Ecology of Apple Fruit and Cider

Fruits are characterized by a predominant microflora that varies from a fruit to another and may be affected by the environment, the geographical area, the weather conditions, the pesticides, or the conditions under which the fruit was developed [22,23,24]. For cider, the perception of “territoriality” is necessary for its appreciation. Its sensory profile is expressively related to microbial activities, and indigenous microorganisms may dynamically contribute to the expression of cider authenticity. Ciders’ microbial ecology comprises numerous genera, species, and strains of bacteria and yeasts. A few studies described the microbiological diversity of fresh apple fruit, apple juice, and cider. Teixidó et al. [25] found that the main microbiota of “Golden Delicious” fresh apples were fungi, such as *Cladosporium* and *Alternaria*, and yeasts. Later, Abadias et al. [26] investigated the same apple’s variety throughout the production and identified low levels of bacteria of the family *Enterobateriaceae* (*Pantoea*, *Citrobacter*, *Enterobacter*, *Klebsiella*, and *Escherichia*). Numerous genera of yeasts such as *Candida*, *Cryptococcus*, *Debaryomyces*, *Kloeckera*, *Kluyveromyces*, *Pichia*, *Rhodotorula*, *Saccharomyces*, and *Zygosaccharomyces* could be also present in fresh fruits [27,28]. Graça et al. [29] found that fungi (yeasts and molds) ranged from 3.6 to 7.1 log CFU/g (colony-forming unit/g) of fresh-cut apples. Most of the isolates obtained in their study were from strains of *Candida sake* and *Pichia fermentans*. *Hanseniaspora* spp., *Candida* spp., *Meyerozyma guilliermondii*, *Metschnikowia pulcherrima*, *Cryptococcus* spp., and *Cystofilobasidium infirmominiatum* were found at lower percentages. Furthermore, mesophilic and psychrotrophic microorganisms were found on fresh apples at a range of 2 to 8.9 and 1.7 to 8.4 log CFU/g, respectively [29,30]. Bacteria were also found on fresh apples in high numbers. Lactic acid bacteria were detected at a range of 1.7–8.7 log CFU/g [29,30]. However, a low number of acid-tolerant bacteria, usually *Acetomonas* species, is frequently present [31].

When it comes to spontaneous cider fermentation, several research studies have shown that the genus *Saccharomyces* is usually predominant. The non-*Saccharomyces* genera, such as *Kloeckera*, *Candida*, *Pichia*, *Hansenula*, *Hanseniaspora*, and *Metschnikowia*, are mostly growing during the first stages of fermentation [24]. The first group of species, which is characterized by a high metabolism includes *Saccharomyces bayanus* and *Saccharomyces cerevisiae* [32], whereas the second one can be classified into two categories: Apiculate yeast, having a low fermentative activity (*Hanseniaspora valbyensis*, *Hanseniaspora uvarum*, and *Hanseniaspora osmophila*), and species primarily showing an oxidative metabolism (*Metschnikowia pulcherrima* and *Pichia guillermondii*) [22,24,33]. The non-*Saccharomyces* yeasts are mainly present during the first stages of fermentation and have a low fermentative capacity [34], while ethanol-tolerant *S. cerevisiae* species are mainly detected in the middle and final stages [35]. A novel species of *Sporobolomyces sucorum* sp. nov. was isolated in apple must and was closely related to *Sporobolomyces pararoseus* and *Sporobolomyces patagonicus* [36]. Some studies showed that the presence of *Saccharomyces* is not common in the must and it is related to the surfaces and production equipment [24,37]. Suárez Valles et al. [38] justified the absence of *Saccharomyces* yeasts in the must due to the usage of a fast pressing system. Moreover, Al Daccache et al. [39] reported that *Hanseniaspora* sp. was the major yeast strain during spontaneous fermentation of “Ace spur” apple juice.

Besides fungi, different genera of bacteria were detected during cider fermentation. Little is known about microbial diversity and physiology of malolactic fermentation (MLF) in cider production. The obligate homofermentative *Lactobacillus mali* [40], *Lactobacillus delbrueckii* subsp. *lactis* [41,42], and *Lactobacillus acidophilus* [43] are noticeably rare. In contrast, the most frequently found species are the heterofermentative lactobacilli: *Lactobacillus collinoides* [44,45,46,47,48], *Lactobacillus paracollinoides* [49], *Lactobacillus fermentum* [44,50], *Lactobacillus buchneri*, *Lactobacillus viridescens, Lactobacillus hilgardii* [44], *Lactobacillus diolivorans*, *Lactobacillus plantarum*, and *Lactobacillus suebicus* [51,52]. Even though some similarities with wine have been well recognized since a long time ago [31,53,54], Sánchez et al. [55] noted that the bacterial species and percentages are different in cider. In their work, a culture-based approach was used to study the diversity of lactic acid bacteria (LAB) by molecular tools. The involved microbiota throughout the MLF were *Lactobacillus*, *Oenococcus*, and *Pediococcus. L. collinoides* was present and predominant throughout the entire process, its distribution alternated with other species such as *Oenococcus oeni* and *Pediococcus parvulus*. However, *P. parvulus* cannot conduct the MLF alone and played an important role in adding flavor intensity to the final product.

## 3. Cider-Making Process

**Figure 1:**
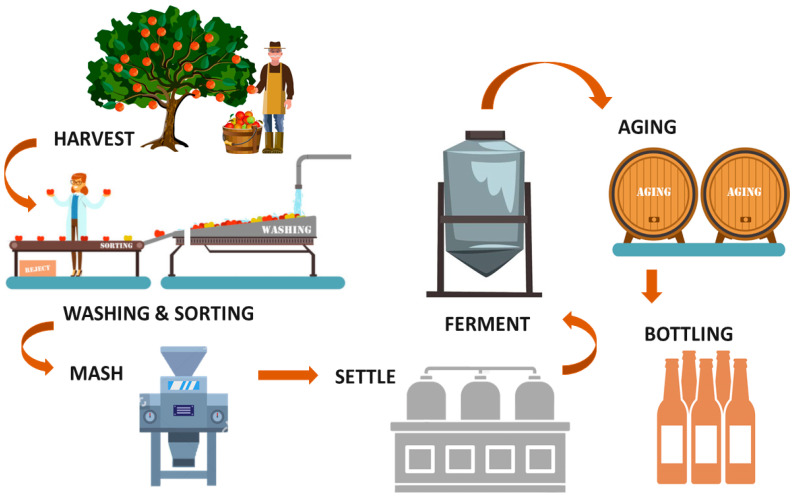
Main steps of the cider-making process.

Different types of ciders exist in the market since every country has its specialty to produce traditional ciders. French cider is usually produced following a natural process without additives or other modern treatments, compared to the English cider. Due to the different production methods, French cider tends to be fruity while the English one is richer in alcohol. Even if the processes seem to be different, many key steps are common to all of these processes (Figure 1).

Apples are first transported from the silo to be machine-washed in water. They are sorted by appearance to remove rotten fruits. The remaining apples are transferred for milling where they are crushed into small pieces. In the French cider process, the apple pulp is oxidized from 30 min to up to 5 h. The pulp is then pressed and left to settle. The fermentation step, which in France relies on natural flora, begins with an oxidative phase. Oxygen flow is highly beneficial for this flora at the beginning of fermentation, leading to limited growth of *Saccharomyces* during this step. This stage is considered very important because this is when fruity aromas are generated. The fermentation is conducted later by *Saccharomyces* for 1 to 3 months at a moderate agitation speed. As for wine production, malolactic fermentation can occur due to bacterial growth in cider. Maturation is the next step after fermentation when other yeasts, such as *Brettanomyces anomalus* can grow, which can have a negative influence on the aromatic quality of the cider. Later, a post-fermentation clarification step takes place, leading to a clear product without turbidity and deposits, and which stabilizes the cider and eliminates haziness caused by the action of proteins or tannins. This step can also eliminate microorganisms and ensure better bacterial stability in the final product. Clarification is done either by settling, centrifugation, or filtration. Finally, after blending and final filtration, the cider is bottled with either carbonation or additional yeast to trigger a second fermentation in the bottle.

Some research works have been conducted to investigate the impact of power ultrasound and pulsed electric fields (PEF) on apple juice fermentation for cider production. Ultrasound- and PEF-assisted fermentations [56,57] showed that the treatment of the yeast strain *Hanseniaspora* sp., isolated from a spontaneous fermented Lebanese “Ace Spur” apple juice [58], may contribute to shortening the fermentation time and to reducing the ethanol content in the fermented product, depending on the parameters applied. Further investigations are, nonetheless, required to study the impact of these emerging technologies on the sensory properties on cider.

## 4. Impact of Apple Juice Composition and Microbial Diversity on Alcoholic Fermentation in the Cider Production Process

**Figure 2:**
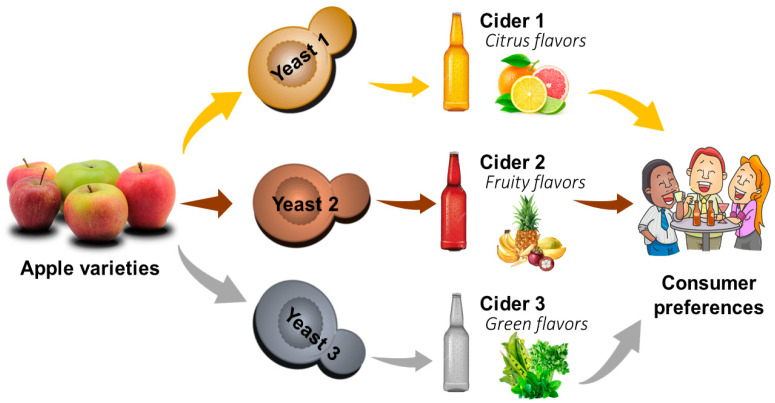
Impact of apple varieties and yeast type on cider production.

Fermentation is a complex metabolic process when sugars are transformed into ethanol, secondary metabolites, acids, alcohols, esters, and carbon dioxide. This transformation can be affected by several parameters related to the fermentation medium. Thus, the choice of apple varieties, as well as the yeast species carrying out the fermentation process, is important (Figure 2).

### 4.1. Impact of Apple Juice Composition on Fermentation

Sugar, acids, and polyphenols represent the three major compounds that affect apple juice fermentation [59]. Accordingly, apple selection is an important step, having a direct impact on the quality of the final product. In countries with ancient cider traditions, special varieties of apples known as “apple cider” are grown for their high levels of acids and phenolic compounds. However, nowadays, dessert apples are more and more frequently used, especially in Germany, Switzerland, and America. Consequently, in order to help cider producers to obtain an optimal mixture, acidity ratios, polyphenols, and alcohols derived from sugars or residual sugars in their products, a quantitative classification system was developed by Long Ashton Cider Research station in the UK [59]. Phenolic compounds have an important effect on the sensory properties of cider such as color, bitterness, and astringency balance, which provide the mouthfeel of cider [59,60]. The phenolic profile may differ from one apple variety to another, but it may also depend on the year of harvest, variety, climate, maturity, storage, and processing [61,62,63,64]. Procyanidins, composed of high molecular compounds, play a major role in astringency, while molecules of lower weights are responsible for bitter taste. In addition, polyphenols can influence the sweetness and acidity, thus affecting overall aroma development during fermentation [65,66]. Not only nonvolatile phenolic compounds play a major role during fermentation, but also the volatile phenolic compounds formed by enzymatic reactions during fermentation contribute to the formation of the aromas of the final product. Another factor to consider is the composition and the concentration of the initial sugars. The nature of the sugar can also affect the fermentation process. Monosaccharides can produce carbon dioxide faster than disaccharides. Furthermore, many other factors can play a role in the progression of fermentation. The glucose and fructose concentrations may influence the yeast growth, i.e., a high sugar concentration will reduce the growth rate of certain yeast strains. For sugar concentrations between 200 and 300 g/L, a decrease in the growth rate of *S. cerevisiae* was observed [67,68]. Furthermore, high sugar levels increase the yeast demand for assimilable nitrogen, which can similarly inhibit the fermentation [69]. For low glucose concentrations, yeasts use sugars either by respiration or fermentation. Aeration induces an increase in the biomass formed (total and per unit of degraded sugar), and at the same time, a decrease in alcohol production and sugar consumption; Pasteur then retained that respiration inhibits fermentation. For high concentrations of glucose, *S. cerevisiae* metabolizes sugars only by fermentation. Even in the presence of oxygen, respiration is impossible. Al Daccache et al. [39] reported different fermentative behaviors of the yeast *Hanseniaspora* sp. during the fermentation of Lebanese “Ace spur” and French “Kermerrien” apple juices. The apples used had different chemical compositions, where the “Ace spur” apple juice had almost the double concentrations of sugars, compared to “Kermerrien” one. Different biomass and ethanol kinetics were obtained. In the presence of an excess of sugar, the yeast cells followed the fermentative pathway from the first hour of fermentation. For the fermentation of “Kermerrien” apple juice, the cells were in a respiratory mode generating biomass in the early hours of fermentation [39]. Some variables, such as temperature and pH, can influence yeast growth rates and the ecology and adaptation of yeast strains [67,68]. Rosend et al. [70] studied the impact of four apple varieties grown in Estonia, Antei, Melba, Kulikovskoye, and Orlovski Sinap, on cider fermentation. Alcoholic fermentation was carried out using the must from the apples at various stages of ripening (i.e., unripe, ripe, overripe) and commercially available yeast strains. The differences in volatile composition between the samples were assessed. The results showed that apple variety stands as the principal attribute influencing the quality and aroma properties of apple cider. The maturity of the fruit was variety-specific, the volatile profiles of Melba variety ciders were the least affected by the ripening stage of apples [70]. Organic acids are indicators of quality during cider fermentation. The dominant flavor of organic acids is sourness, but they also contribute to bitterness and astringency of cider [71]. Some yeasts can assimilate malic acid resulting in its reduction, fluctuating from 5 to 40% [72]. When a second bacterial fermentation occurs, its level is reduced mainly by lactic acid bacteria. During this fermentation, citric acid is transformed into acetic acid, whereas shikimic and quinic acids are metabolized to single phenols, like catechol and ethylcatechol, and other compounds. Organic acids may affect the yeast metabolism. The yeast enzymatic activity and the chemical alterations are also influenced by the juice acidity [73].

### 4.2. Impact of Yeast on Fermentation

Yeast plays an essential role in the production of all alcoholic beverages, and the selection of an appropriate yeast strain is crucial to control the alcohol yield and to preserve the beverage’s sensory quality. Fermentative yeasts can use sugars anaerobically as electron donors, electron acceptors, and carbon sources. However, the yeast action during fermentation is not only limited to the transformation of sugars into alcohol. Yeast metabolism produces different other metabolites and by-products that may have an essential impact on the organoleptic quality of the fermented product [74]. Thus, the criteria to select yeast strains for their use in fermented beverages comprise their capability to dominate the media and to improve desired sensorial characteristics and their inability to produce undesired compounds such as biogenic amines or off odors [75]. During spontaneous fermentation, several yeast species may be present and could play a significant, complex, and unpredictable role [76]. Some yeast species may be present only during the first stage of fermentation, while others, more resistant to ethanol, are dominant during the later stages. This type of yeast is nowadays known as belonging to the *Saccharomyces* strains [77]. *S. cerevisiae* is largely used to produce alcoholic beverages due to its controlled and repetitive behavior as well as for the release of its aroma precursors [78,79,80,81]. Nevertheless, fermentation is the collaboration of different species of yeast and bacteria initially present in the product or found on the surface of the presses and fermenters. Mixed fermentations are suggested as a feasible way to improve the complexity and enhancing the particular and specific characteristics of the product [82]. The growth of each yeast species is characterized by a definite metabolic activity, which determines the concentrations of flavor compounds in the final product. Therefore, the role of non-*Saccharomyces* yeasts appears important during the fermentation process. The main yeasts present in the early stages of fermentation belong to the genera *Hanseniaspora* and *Candida*. These species are characterized by a low fermentation capacity and are sensitive to an alcohol concentration close to 5 or 6%. In addition, some changes in fermentation parameters may result in the presence of yeasts such as *Brettanomyces*, *Kluyveromyces*, *Schizosaccharomyces*, *Torulaspora*, *Zygosaccharomyces*, and *Saccharomycodes* [83,84,85]. From the above-cited yeasts, some of them may have a positive impact on fermentation by releasing favorable aromas, but others may release undesirable aromas known as off-flavors. Yeasts can affect primary aroma determined by the initial composition of the product and the secondary aromas that are created during the fermentation, as well as the tertiary aromas generated during the maturation of the finished product [86]. *Hanseniaspora*, *Zygosaccharomyces*, and *Schizosaccharomyces pombe* species produce high amounts of volatile fatty acids, such as acetic acid [87,88,89,90,91], and low concentrations of higher alcohols [92,93,94,95]. Esters and sulfur compounds are mainly produced by *Candida*, *Hansenisapora*, *Torulaspora delbrueckii*, and *Kazachstania gamospora* [93,96,97,98]. Lorenzini et al. [99] investigated the capacity of *Torulaspora delbrueckii*, *Hanseniaspora osmophila*, *Hanseniaspora uvarum*, *Starmerella bacillaris*, and *Zygosaccharomyces bailii* to ferment apple juice and found that *Hanseniaspora uvarum* was the greatest producer of hexyl and isoamyl acetate. The complex volatile profile of cider suggests the possible strain-specific effects on the aroma formation. Wei et al. [100] tried to enhance the flavor complexity of cider by different non-*Saccharomyces* species. The chemical composition and sensory properties of five different fermentations of mixed cultures of *Pichia kluyveri*, *Hanseniaspora vineae*, *Hanseniaspora uvarum*, and *Torulaspora quercuum* were studied for apple juice fermentation. The results indicated that the growth of *P. kluyveri* and *H. vineae* were interreacted and affected by *H. uvarum* and *T. quercuum*. Furthermore, *H. vineae* was able to consume more sugar than *P. kluyveri*. In general, the fermentations involving *H. uvarum* displayed high pH values, whereas those involving *P. kluyveri* and the mixed *P. kluyveri* and *H. uvarum* resulted in high levels of residual sugar, sugar/acid ratio, and glucose-fructose consumption ratio. The pair *P. kluyveri* and *H. uvarum* produced the highest concentration of glycerol. Noticeable variations in organic acids and polyphenols were observed between the different fermentations. The analysis showed that acetate esters contributed the most positively to the roasted and cooked aroma note in all ciders. This was the first study evaluating the simultaneous fermentation of two non-*Saccharomyces* yeasts to produce cider. A recent study described the antagonistic and fermentative properties of *Starmerella bacillaris.* The yeast proved to positively modulate cider volatile profile in the microfermentation trials [101]. *Brettanomyces*, *Kluyveromyces*, *Schizosaccharomyces*, *Torulaspora*, *Zygosaccharomyces*, and *Saccharomycodes* have a negative influence on the product [102]. *Brettanomyces* may produce 2-ethyltetrahydropyridine, 2-acetyltetrahydopyridine, and 2-acetylpyrroline, causing taste defects and unpleasant smell in beverages.

Non-*Saccharomyces* yeasts have high enzyme activity such as β-glucosidase, esterase, and β-lyase. This enzyme activity contributes to a higher concentration of terpenes and thiols that may add a positive fruity aroma and fragrance to the fermented product [103,104,105,106]. De Arruda Moura Pietrowski et al. [107] and Wosiacki et al. [108] noted that the strains of *Hanseniaspora* sp. have a positive impact on the aromatic profile of cider, thereby accentuating the beneficial role of these yeasts. Nowadays, modern oenology is searching for novel strategies to reduce the final ethanol content in fermented beverages. This trend is due to consumer demand for products with lower ethanol content. The use of non-*Saccharomyces* species reduces the initial ethanol content by approximately 1–2% (*v*/*v*), depending on the yeast species and fermentation conditions [109,110,111]. In addition, these yeasts can be used to regulate the acidity of drinks [112,113] as *Saccharomyces* yeasts have no significant influence on acidity [114,115], and conventional chemical methods consist of the addition of expensive and qualified products being of food quality.

## 5. Conclusions

This review emphasized the apple physicochemical and microbial composition and showed how the fermentation can be affected by the first material composition. The present review also leads the way for the optimization of the apple fruit fermentation by controlling the composition of the raw material. In addition, the review underlined the importance of the microbial ecosystem of musts and exposed how mastering the quality and the safety of cider production are reliant on a better understanding of the mechanisms and yeast metabolism.

## Figures and Tables

**Table 1:** Global production and consumption of fresh apples per year in 2019 [2].

Country	Apple Production (kt)	Fresh Domestic Consumption (kt)
China	33,000	38,050
European Union	15,442	7400.6
United States	5564	2589.4
Turkey	3306	2630.5
Iran	3085	1813.9
Russia	1656	1884.4
Chile	1393	229.6
Ukraine	1211	1066.2
Brazil	1156	1325.9

**Table 2:** Simple and parietal sugar contents of apple fruit [8,9].

Compounds	Concentrations
Glucose *	1.8
Fructose *	5.6
Sucrose *	2.6
Arabinose **	109
Rhamnose **	12
Fucose **	8.5
Galactose **	71.5
Glucose **	288
Mannose **	21
Xylose **	57
Galacturonic Acid **	227

* g/100 g of fresh fruit; ** mg/g of the cell wall materials.

**Table 3:** Average content of phenolic compounds in apple flesh and peel (single compounds and total concentrations are expressed in mg/100 g freeze-dried material, TPC denotes total phenolic compounds and is expressed in mg GAE/100 g freeze-dried material) [11]. “n.d.” denotes “not detected”, “GAE” denotes “Gallic Acid Equivalent”.

Compounds	Apple Flesh	Apple Peel
Procyanidin B1	1.0 ± 0.1	1.9 ± 1.6
(+) -catechin	1.1 ± 0.9	5.1 ± 3.0
Procyanidin B2	1.6 ± 0.3	7.1 ± 1.5
Procyanidin C1	n.d.	6.7 ± 1.2
(−) -epicatechin	1.0 ± 0.7	8.8 ± 4.9
Procyanidin A2	2.5 ± 1.2	7.8 ± 2.9
**Total Flavanols**	6.4 ± 2.5	36.1 ± 8.8
Gallic acid	1.6 ± 0.2	
Protocatechuic acid	0.1 ± 0.0	1.3 ± 0.6
Chlorogenic acid	2.1 ± 0.8	5.6 ± 0.7
Caffeic acid	0.8 ± 0.5	0.8 ± 0.5
P-coumaric acid	0.5 ± 0.1	1.8 ± 0.4
Ferulic acid	0.1 ± 0.1	0.9 ± 0.4
**Total Phenolic Acids**	5.2 ± 1.3	14.3 ± 2.1
Phloridzin	1.1 ± 0.7	4.8 ± 3.5
Hyperoside	n.d.	84.2 ± 57.1
Isoquercitrin	n.d.	16.6 ± 8.6
Rutin	n.d.	5.4 ± 3.3
Reynoutrin	n.d.	17.0 ± 7.6
Avicularin	n.d.	21.4 ± 8.4
Quercitrin	n.d.	25.4 ± 13.1
Quercetin	n.d.	13.4 ± 5.2
**Total flavonols**	n.d.	183.5 ± 99.6
**Total polyphenols**	12.6 ± 4.4	239.4 ± 118.6
**TPC**	179.5 ± 52.3	914.7 ± 331.3

**Table 4:** Lipids content of apple flesh [8].

Compounds	% of Total Lipids
Triacylglycerols	5
Glycolipids	17
Phospholipids	47
Sterols	15
Sterol esters	2
Sulfolipids	1
Others	13

**Table 5:** Mineral contents in apple [8].

Minerals	mg/100 g Dry Matter
Potassium	840
Sodium	7.9
Calcium	38
Magnesium	40
Iron	1.6
Aluminum	0.43
Phosphorus	73
Zinc	0.65
Manganese	0.3
Copper	0.35
